# Characterization and Seasonal Modulation of Adenosine A_1_ Receptors in the Arctic Ground Squirrel Brain

**DOI:** 10.3390/ijms24021598

**Published:** 2023-01-13

**Authors:** Zachary Carlson, Kelly Drew

**Affiliations:** 1 Institute of Arctic Biology, University of Alaska, Fairbanks, AK 99775, USA; 2 Center for Transformative Research in Metabolism, University of Alaska, Fairbanks, AK 99775, USA

**Keywords:** hibernation, torpor, adenosine, adenosine A_1_ receptor, arctic ground squirrel, N^6^-cyclohexyl adenosine, seasonal sensitization

## Abstract

Hibernation is an adaptation that allows animals such as the Arctic ground squirrel (AGS) to survive the absence of food or water during the winter season. Understanding mechanisms of metabolic suppression during hibernation torpor promises new therapies for critical care. The activation of the Adenosine A_1_ receptor (A_1_AR) has been shown to be necessary and sufficient for entrance into hibernation with a winter season sensitization to the agonist, but the role of the A_1_AR in seasonal sensitization is unknown. In the current study, we characterize the A_1_AR in the forebrain, hippocampus and hypothalamus of summer and torpid AGS. For the first time, we define the pharmacological characteristics of the A_1_AR agonist, N^6^-cyclohexyladenosine and the A_1_AR antagonist dipropylcyclopentylxanthine (DPCPX) in the AGS brain. In addition, we test the hypothesis that increased A_1_AR agonist efficacy is responsible for sensitization of the A_1_AR during the torpor season. The resulting ^35^S-GTPγS binding data indicate an increase in agonist potency during torpor in two out of three brain regions. In addition to ^35^S-GTPγS binding, [^3^H]DPCPX saturation and competition assays establish for the first-time pharmacological characteristics for the A_1_AR agonist, N^6^-cyclohexyladenosine and the A_1_AR antagonist dipropylcyclopentylxanthine (DPCPX) in AGS brain.

## 1. Introduction

Torpor is a fundamental metabolic state of energy conservation. Hibernation, defined by prolonged torpor lasting days to two or more weeks, interrupted by brief 12–24 h episodes of euthermic metabolism, represents some of the most extreme examples of mammalian energy conservation. Evidence suggests that hibernation is an extension of sleep that involves the modulation of thermogenesis [1]. In ground squirrels, suppression of thermogenesis is sufficient to account for the initial fall in metabolic rate, after which a decline in core body temperature suppresses metabolic rate further through thermodynamic influence on metabolic processes [2]. Adenosine, a ubiquitous neuromodulator present in most tissues, modulates thermogenesis in the CNS [3,4]. The purine binds to four subtypes of G-protein coupled receptors, designated the adenosine A_1_, A_2A_, A_2B_ and A_3_ receptors (A_1_AR, A_2_aAR, A_2b_AR, and A_3_AR, respectively). In the brain, A_1_AR and A_2A_AR are widely expressed with only a small amount of A_3_AR, whereas A_2B_AR is only present in peripheral tissues. A_1_AR and A_3_AR receptors propagate their signal through G_i/o_ G-proteins and are neuroinhibitory. In contrast, the A_2A_AR and A_2B_AR interact with G_s_ proteins and are neuroexcitatory. 

Stimulation of A_1_AR within the CNS is necessary for the onset of torpor in hibernating Arctic Ground Squirrels (AGS; *Urocitellus parryii)*; however, the response is regulated by a process entrained to a circannual rhythm that governs seasonal sensitivity to the A_1_AR agonist N^6^-cyclohexyladenosine (CHA). Evidence from our laboratory suggests that the winter season enhances the influence of CHA on thermogenesis within the median preoptic nucleus and the rostral raphe pallidus to inhibit the premotor sympathetic neurons within the rPA that drive thermogenesis [5]. The mechanism underlying seasonal sensitivity to CHA is unknown but may involve changes in adenosinergic tone within thermoregulatory nuclei in the hypothalamus or changes at the level of the A_1_AR.

Sleep and thermogenesis are modulated, in part, by A_1_AR [6] and A_2_AR [7]. Hibernation, sleep and thermogenesis show seasonal rhythms in ground squirrels [1,8] and humans [9], but it is unknown if the properties of A_1_AR agonists or the expression of A_1_AR vary with season. In the current study, we establish for the first-time pharmacological characteristics of the adenosine agonist CHA and antagonist DPCPX in AGS brain tissue. In addition, we test the hypothesis that a seasonal shift in the potency of CHA, the A_1_AR agonist most studied regarding A_1_AR agonist-induced hibernation and torpor, is associated with the seasonal expression of hibernation in AGS. 

## 2. Results

**Figure 1:**
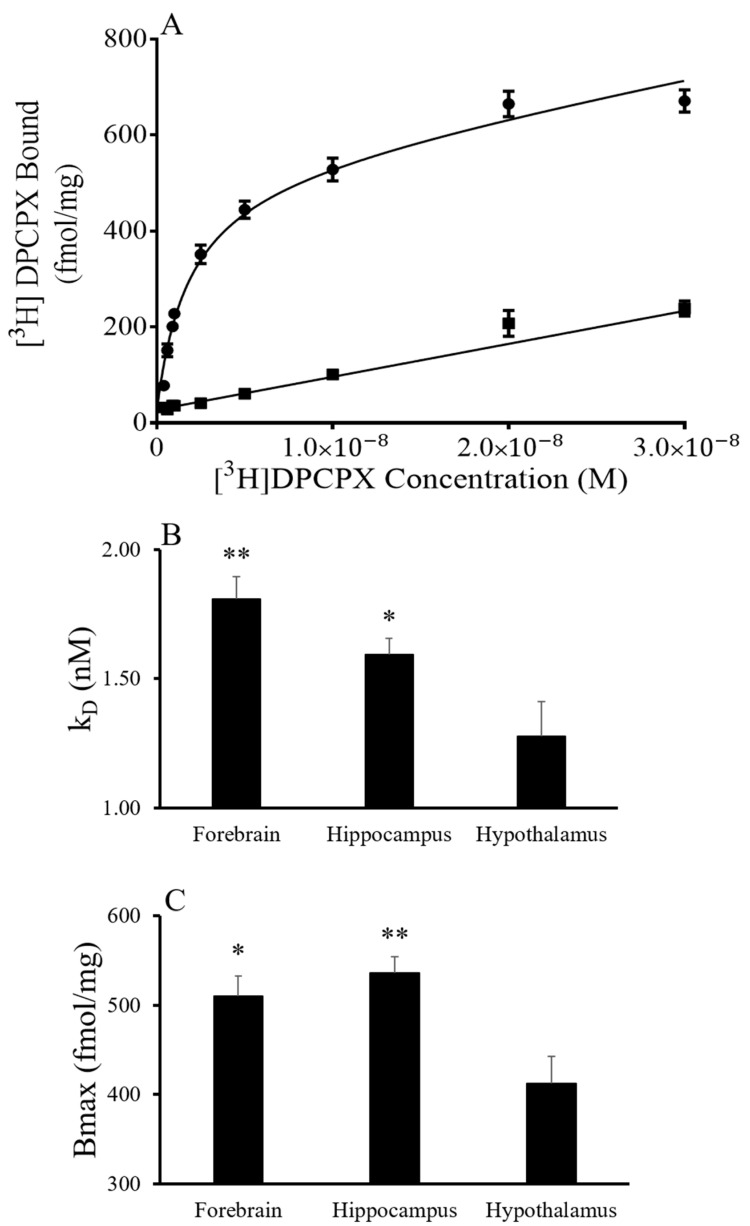
Kinetics of [^3^H] DPCPX binding in AGS with results from summer and torpid groups combined. Binding of [^3^H] DPCPX in the forebrain was similar in appearance to that seen in the hippocampus and hypothalamus (**A**). [^3^H] DPCPX demonstrated nanomolar affinity for the A_1_AR in the three tissues tested. The affinity of [^3^H] DPCPX binding was lower in the hypothalamus than in the other brain regions (**B**). Also, the number of A_1_AR, indicated by Bmax, in the hypothalamus during the torpid season was less than in the forebrain and hippocampus (**C**) ** *p* < 0.01, * *p* < 0.05 vs. hypothalamus, Tukey, n = 4–8.

To characterize the A_1_AR, saturation and displacement assays were conducted in the forebrain, hippocampus and hypothalamus of AGS euthanized during the summer and torpid state in the winter season. Saturation experiments using [^3^H] DPCPX indicated a single site model and yielded K_D_ and Bmax values that showed an effect of brain region but not of the season (Figure 1). The saturation curves for [^3^H] DPCPX binding were similar in the forebrain, hypothalamus and hippocampus of tissue collected in summer and from torpid AGS in winter (Figure 1). The results indicate that the hypothalamus has a lower K_D_ and Bmax than the forebrain and hippocampus independent of the season (*p* = 0.0017, two-way ANOVA, the main effect of the region for K_D_; *p* = 0.0022, the main effect of the region for Bmax). 

**Figure 2:**
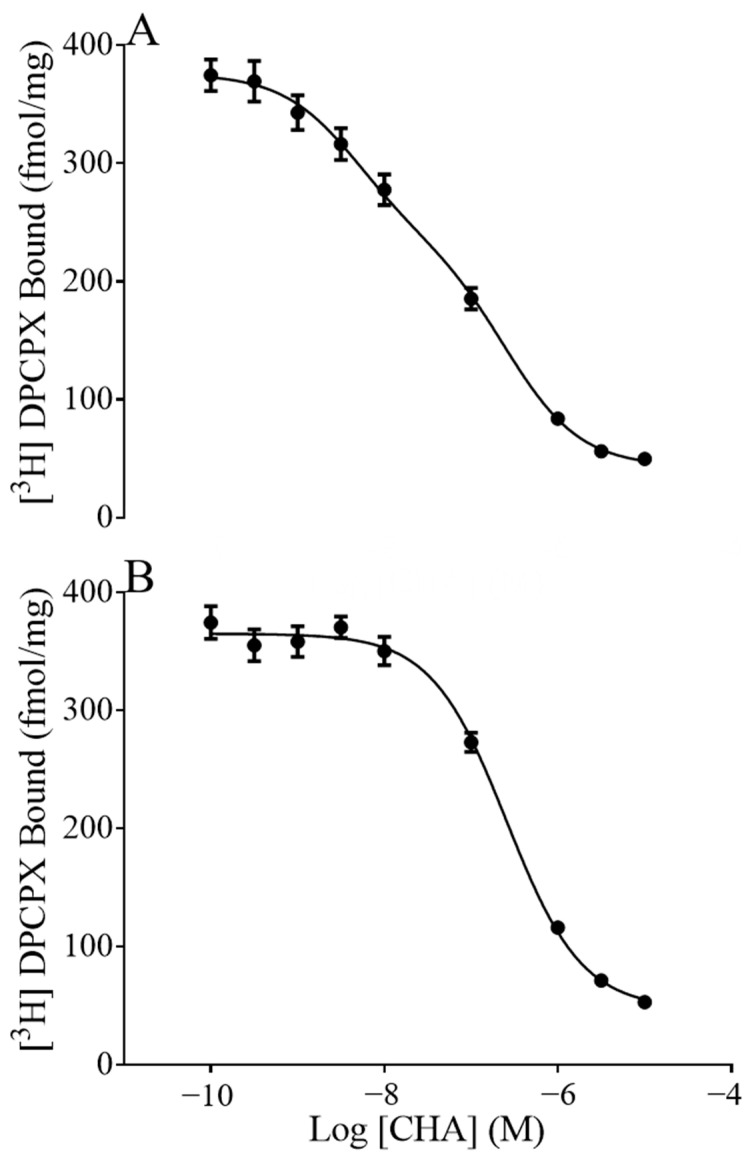
[^3^H] DPCPX displaced with CHA in the presence or absence of GTP. Summer forebrain binding curves were best fit by a two-site model in the absence of 1 µM GTP (**A**) and one-site in the presence of 1 µM GTP (**B**). The general characteristics of these graphs were conserved in the winter season of the forebrain as well as the hippocampus and hypothalamus (not shown).

To ask if the fraction of receptors in the high or low affinity states was associated with seasonal sensitivity to CHA, we displaced [^3^H]DPCPX with CHA. As expected, displacement assays fit a two-site model indicating the presence of a low and high affinity site (pki_Hi_,Pki_Lo_). Furthermore, in the presence of GTP, only the pki_Lo_ site was detected, as high concentrations of GTP promote the disassociation of G-proteins from GPCRs (Figure 2). As expected, the pki_Lo_ established by the two-site model did not differ from the GTP shift pki_Lo_, arguing that the GTP shift did indeed isolate the low affinity site. Here we found that the fraction of receptors in the high affinity state (Fraction High) was greater during torpor than during summer (*p* = 0.0071, two-way ANOVA, main effect of season, Table 1). In addition, there was an effect of region on the affinity of CHA at the low affinity site where the pki_Lo_ in the hippocampus was significantly less than the pki_Lo_ in the forebrain and hypothalamus, regardless of season (*p* < 0.0001, two-way ANOVA, the main effect of region, with post-hoc Tukey *p* < 0.0001 hippocampus vs. forebrain; *p* < 0.0001 hippocampus vs. hypothalamus). The ratio of dissociation constants at the high and low affinity sites (pki_Hi_/pki_Lo_) was also greater in hippocamps than in other regions (*p* < 0.001 two-way ANOVA, main effect of region). Post-hoc analysis showed that the hippocampus differed from the hypothalamus (*p* < 0.001, Tukey) and trended towards being different from the forebrain (*p* = 0.094).

**Figure 3:**
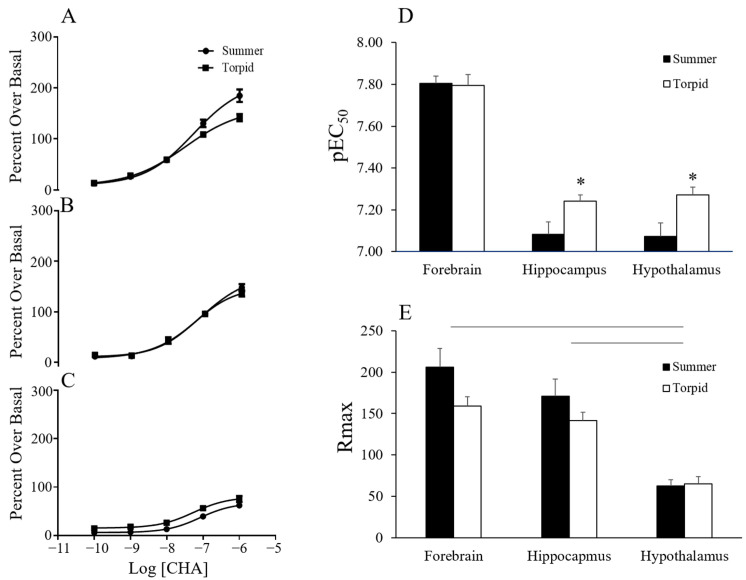
CHA-induced ^35^S-GTPγS binding in summer and torpid AGS brain tissue. Three brain tissues were analyzed: forebrain (n = 6, 5; summer, torpid) (**A**), hippocampus (n = 7, 7; summer, torpid) (**B**) and hypothalamus (n = 7, 6; summer, torpid) (**C**). (**D**) An increase in pEC_50_ indicates an increase in the potency of CHA in the hippocampus and hypothalamus in torpid vs. summer AGS; * *p* < 0.05, *t*-test. Horizontal lines in (**E**) indicate differences between groups, *p* < 0.05.

**Figure 4:**
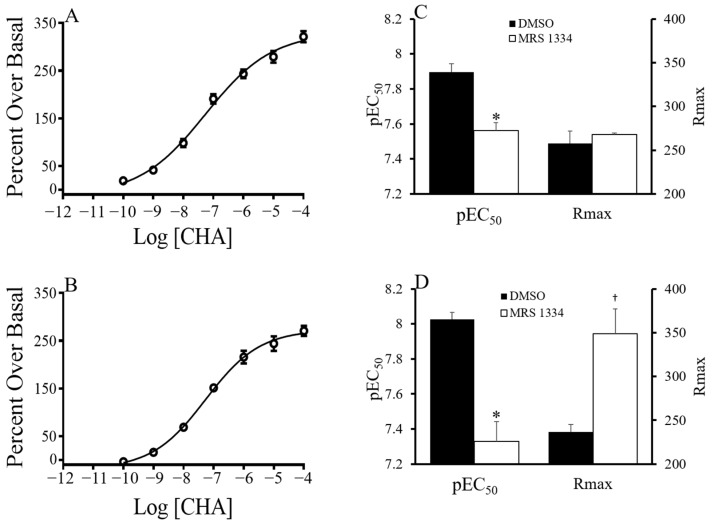
CHA (100 pM to 100 µM)-induced GDP/GTP exchange measured by ^35^S-GTPγS binding in the presence of A_3_AR antagonist, MRS 1334 dissolved in DMSO. Inhibiting A_3_AR had no effect on the Hill slope of CHA-induced GDP/GTP exchange in the forebrain of summer (hillslope = 0.55 ± 0.042, 0.66 ± 0.024, n = 3, 3; DMSO, MRS 1334) (**A**) or torpid (hillslope = 0.61 ± 0.56, 0.56 ± 0.042, n = 3, 3; DMSO, MRS 1334) AGS (**B**), showing that the low Hill slope was not due to CHA binding to the A_3_AR at higher concentrations. MRS 1334 reduced the potency of CHA in summer (**C**) and torpid (**D**) tissues. In torpid tissue, preincubation with MRS 1334 increased Rmax when compared with DMSO (**D**). * *p* < 0.01, † *p* < 0.05 vs. DMSO, *t*-test.

The functional response of CHA was investigated by agonist-induced ^35^S-GTPγS binding. Concentrations of CHA up to 1 µM stimulated ^35^S-GTPγS binding in a single-site manner. The resulting data revealed an effect of season and brain region on the pEC50 as well as an effect of region on the maximum signal (Rmax) (Figure 3). CHA was more potent in tissue collected during torpor than during summer (*p* < 0.005, two-way ANOVA, the main effect of the season). A trend towards an interaction between region and season (*p* < 0.091) led us to perform further one-way analyses over the season that showed increased potency of CHA in torpor in the hypothalamus (*p* < 0.05, *t*-test) and in the hippocampus (*p* < 0.05, *t*-test) but not in the forebrain (Figure 3). The Rmax was greatest in the forebrain and hippocampus and lowest in the hypothalamus (*p* < 0.0001 two-way ANOVA, the main effect of region, with post-hoc Tukey *p* < 0.0001 hippocampus vs. hypothalamus; *p* < 0.0001 hypothalamus vs. forebrain). During the characterization of the functional response of CHA, a low hillslope was observed at concentrations greater than 1 µM. A low hillslope is indicative of negative cooperativity or a second lower affinity binding site. CHA has been shown to have a low affinity for the A_3_AR in the rat brain [10], and therefore A_3_AR could be stimulated by high concentrations of CHA. We hypothesized that CHA at concentrations above 1 µM would stimulate the A_3_AR resulting in a low hillslope. To eliminate A_3_AR binding, we repeated the experiment in the presence of an A_3_AR antagonist (MRS 1334; [11]. In AGS, summer and torpid forebrain tissue, pre-blocked with 500 nM of MRS 1334, was stimulated with up to 100 µM CHA and ^35^S-GTPγS binding was measured (Figure 4). The inclusion of MRS1334 did not have an effect on the hillslope of CHA-induced ^35^S-GTPγS binding in either season, providing evidence that the A_3_AR stimulation was not contributing to the low hillslope. The potency of CHA, as indicated by the pEC_50_, decreased in the presence of MRS1334, and the decrease was greater in tissue from torpid ASG than in tissue from summer AGS (*p* < 0.0001, two-way ANOVA, the main effect of treatment; *p* < 0.05 treatment x season). Interestingly, the Rmax only increased in the torpid tissue (*p* < 0.05, *t*-test torpid MRS1334 vs. vehicle).

**Figure 5:**
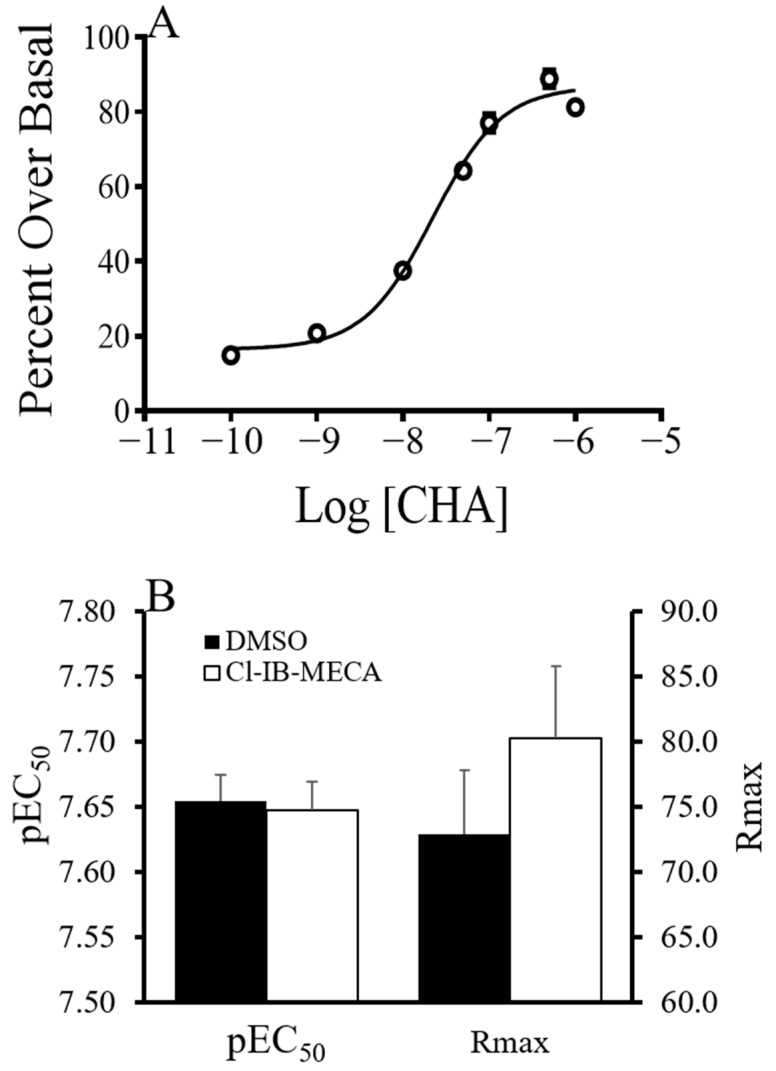
CHA-induced GDP/GTP exchange measure with ^35^S-GTPγS binding in the presence of A_3_AR agonist. Preincubation with Cl-IB-MECA (n = 4, 4; DMSO, Cl-IB-MECA) (**A**) did not decrease the hill slope of the 100 pM–1 µM dose range as would be expected if the A_3_AR stimulation was inducing the low hill slope at higher concentrations of CHA (DMSO hillslope = 1.06 ± 0.0864; Cl-IB-MECA hillslope = 1.08 ± 0.0407). The presence or absence of Cl-IB-MECA had no effect on any pharmacological properties of CHA-induced ^35^S-GTPγS binding (**B**).

It has been well established that GPCRs form dimers and higher order oligomers with GPCRs which can affect agonist signaling. If the effect of blocking A_3_AR on Rmax or pEC_50_ was due to A_1_AR and A_3_AR cross-talk, we asked if stimulating A_3_AR would cause a change in Rmax or pEC_50_. The A_3_AR was stimulated with 132 nM 2-Chloro-N^6^-(3-iodobenzyl)-adenosine-5′-N-methyluronamide (Cl-IB–MECA). CHA-stimulated GDP/GTP exchange in the forebrain of summer AGS was then measured at concentrations up to 1.0 µM CHA using ^35^S-GTPγS binding assay (Figure 5). There was no effect of Cl-IB-MECA on Rmax or pEC_50_.

## 3. Discussion

This study is the first to characterize A_1_AR in the brain of summer and torpid AGS and to identify changes at the receptor level that may contribute to the seasonal shift in sensitivity to torpor-inducing effects of CHA. Results indicate that the potency of A_1_AR agonists, reflected by the EC_50_ in the GTP binding assay, increases in the hippocampus and hypothalamus during torpor when compared to the summer season. However, the shift in potency could not be explained by an increase in affinity or efficacy with the assays used and sample size available from the tissue bank. These findings have implications for the seasonality of sleep and thermoregulation in humans. as well as the seasonal efficacy of therapeutics. 

### 3.1. Characterization of A_1_AR Ligand Binding and Agonist-Induced GDP/GTP Exchange

Direct ligand binding and competition assays have, for the first time, characterized the receptor pharmacology of CHA and DPCPX in AGS brain tissue. As expected, [^3^H] DPCPX saturation experiments produced a one-site binding curve at the A_1_AR [12]. K_D_ for DPCPX was similar to K_D_’s reported in rat brain and smooth muscle preparations as well as sheep pineal membranes [13,14,15]), although the affinity of ligands for adenosine receptors are species dependent [16]. Bmax for DPCPX binding was consistent between brain regions, as would be expected given the ubiquitous distribution of A_1_AR in the brain.

CHA was found to induce GDP/GTP exchange as expected for an A_1_AR agonist, as shown in rat brain tissue. GTP produced a characteristic shift to a single low affinity binding site (Ki_lo_); the magnitude of this shift represented by the ratio of the pKi_Hi_/pKi_Lo_, a measure of agonist efficacy, was similar between seasons in the hypothalamus and hippocampus where potency was found to be greater in torpid/compared to summer. In the forebrain, where potency did not change with the season, we found evidence of an increased abundance of receptors in the high affinity state. A higher fraction of receptors in the high affinity state could indicate a greater proportion of functional receptors coupled to G-protein. Although an increase in efficacy could not explain seasonal differences in potency in the hippocampus or hypothalamus, we saw a clear difference in efficacy between brain regions. The maximal cellular response to agonist-stimulated GDP/GTP exchange, another measure of agonist efficacy, varied between brain regions with more than a 5-fold difference between the highest response in the forebrain and the lowest response in the hypothalamus. 

### 3.2. Potential Interaction between GPCR

A hillslope of less than one was noted at higher concentrations of CHA which was not due to binding at the A_3_AR site, although other binding sites could not be ruled out. Given that the A_2B_AR is not normally expressed in the brain and CHA has a very low affinity for the A_2A_AR, the second site is most likely not another adenosine receptor. The low Hill slope at higher concentrations of CHA could also be due to negative cooperativity that occurs when the A_1_AR forms homomers [17]. 

Interestingly, inhibition of the A_3_AR decreased the potency of CHA independent of season, suggesting positive cross-talk between the A_1_AR and the A_3_AR. However, results argued against cross-talk because stimulating the A_3_AR did not increase the potency of CHA. Alternatively, 500 nM of MRS 1334 may have inhibited the A1 receptor.

In summary, evidence supports a role for increased efficacy of CHA in the hypothalamus and hippocampus during the winter season as a mechanism that may contribute to seasonal sensitivity to CHA. Direct ligand binding and measurements of GDP/GTP exchange in AGS brain tissue yield results consistent with the behavior of A_1_AR ligands in other species. Evidence for a seasonal change in the receptor pharmacology of A_1_AR agonists demonstrates that endogenous rhythms may influence drug-receptor interactions. Similar seasonal influences in humans could have clinical implications for the vast pharmacopeia of GPCR ligands. Although the results reported here cannot explain the mechanism for altered efficacy, further study of the mechanism would have translational significance for developing A_1_AR agonists as therapeutics. The means to increase CNS efficacy would minimize individual differences in response to CHA reported previously [18,19] and potentially decrease peripheral side-effects such as hypotension and bradycardia by decreasing the therapeutic dose of agonist. Although A_1_AR agonists have limited translational potential due to peripheral side-effects, combining a CNS active agonist with an antagonist that does not cross the BBB shows promise as a means to target CNS sites of action [20]. A limitation of the study is that we did not include a winter group that was not torpid. There is a possibility that characteristics of the A_1_AR or CHA activation of the A_1_AR change between interbout arousal and torpor. Although we cannot rule out this possibility, we expect that by using the same assay temperature in summer and winter/torpid tissues, we avoided many of the influences of torpor that could have confounded the interpretation of a seasonal effect. Seasonal alteration of CHA pharmacokinetics or endogenous levels of adenosine may also play a role but were beyond the scope of this study. Translating hibernation for human medicine and defining mechanisms that underly seasonal sleep drive and sensitivity to therapeutics have broad implications for the future of therapeutics.

## 4. Materials and Methods

### 4.1. Arctic Ground Squirrels

AGS tissue was obtained from a tissue bank (courtesy of B. Barnes, Fairbanks, AK, USA). All animal procedures were approved by the UAF Institutional Animal Care and Use Committee (protocol #06-44). AGS were captured near 66°38′ N, 149°38′ W under permit from the Alaska Department of Fish & Game. Animals were housed at 22 °C on an 18:6 day: night cycle (5/2011–8/2011) and at 2 °C on a 4:20 day: night cycle (8/2011-time of tissue collection; 1/2011). Hibernation was monitored using the “shavings added” method, where hibernation (torpor) is indicated when shavings placed on the back of the AGS remain undisturbed 24 h later [21,22]. All tissue was harvested from adult male AGS during the summer season or while torpid during the winter season. The summer season was defined as AGS that were captured after the previous hibernation season and kept in captivity for two months before tissue collection. Tissue from torpid AGS were collected during the winter season, after at least six to eleven torpor bouts and at least ten but not more than thirteen days in the current torpor bout.

Summer AGS were euthanized by decapitation under a surgical plane of anesthesia (isoflurane, 4% mixed with 100% medical grade oxygen, delivered at 1.5 L/min until unresponsive to a toe pinch). Torpid AGS were euthanized without being aroused from torpor and did not require anesthesia nor breathe at a rate sufficient to absorb the gas anesthesia. Immediately following euthanasia, the brain was removed, and the hippocampus, hypothalamus and remaining forebrain were isolated and frozen in liquid nitrogen. All tissue was stored at −80 °C until use.

### 4.2. Isolation of Plasma Membrane for ^35^S GTPγS and [^3^H]DPCPX Binding Experiments

AGS membranes from the forebrain, hippocampus, hypothalamus and brainstem were isolated as described previously with modifications [23]. Briefly, tissue was homogenized on ice using an all glass Dounce homogenizer (10–15 strokes) in 20× volume homogenization buffer containing 10 mM HEPES, 2 IU/mL ADA, 640 mM sucrose and protease inhibitor tablets (Roche, Indianapolis, IN, USA) and then further homogenized by polytron for 10–15 s. The suspension was centrifuged at 1000× *g* for 10 min at 4 °C. Resultant supernatant was centrifuged at 48,000× *g* for 15 min at 4 °C. Pellets were resuspended in Resuspension buffer containing 10 mM HEPES, 2 IU/mL ADA and protease inhibitor tablets. The suspension was centrifuged at 48,000× *g* for 15 min at 4 °C. Pellets of AGS hippocampus and hypothalamus were suspended in a solution containing 6 mM HEPES, 122 µM GDP and 2.4 IU/mL ADA, the forebrain was suspended in 6 mM HEPES, 77 µM GDP, and 0.5 IU/mL ADA and both were incubated at room temperature under gentle rocking for 60 min and then centrifuged at 48,000× *g* for 30 min. Subsequent pellets were resuspended in a Resuspension buffer and stored at −80 °C until use.

### 4.3. [^3^H] DPCPX Binding

To ask if membrane expression of the A1AR increased in the winter season, we performed saturation experiments to determine the K_D_ and Bmax of [^3^H] DPCPX binding to A_1_AR were conducted on the membrane of the forebrain, hippocampus and hypothalamus of winter and summer AGS following the guidelines of (Hulme 2010) with modification [22]. On the day of the experiment, aliquots of summer and torpid AGS were thawed on ice. The protein content of each animal was determined by protein analysis (Bio-Rad, Hercules, CA, USA) followed by centrifugation at 48,000× *g* for 30 min at 4 °C. The pellet was then resuspended in a solution containing 50 mM HEPES and 2 IU/mL ADA. Saturation experiments were performed by incubating 100 µg/mL protein with nine concentrations of [^3^H] DPCPX ranging between 0.4 and 30 nM in the presence of 50 mM HEPES and 2 IU/mL ADA. Non-specific binding was defined in the presence of 7 µM cyclopentyltheophylline (CPT). The solution was allowed to incubate for 90 min at room temperature, and the membrane bound ligand was isolated as described below.

### 4.4. ^35^S-GTPγS Binding

To investigate the functional response of CHA activation of the A1AR in summer and torpid animals, ^35^S-GTPγS binding experiments were performed as described previously with modifications [23]. On the day of the experiment, aliquots of summer and torpid AGS were thawed on ice. The protein content was then determined by protein analysis (Bio-Rad, Hercules, CA, USA) followed by centrifugation at 48,000× *g* for 30 min at 4 °C. The pellet was then resuspended in Assay buffer containing 50 mM HEPES, 200 mM NaCl, 10 mM MgCl_2_, 40 µM GDP, 100 µM Saponin, 1 IU/mL ADA and 1 mM DTT at pH 7.4. 100 µg per ml protein was incubated with 400 pM of ^35^S-GTPγS in a total volume of 100 µL for 90 min under gentle rocking at 37 °C. The non-specific activity was determined in the presence of 5 µM GTPγS. The constitutive activity was defined as binding in the absence of CHA. The reaction was terminated by rapid vacuum filtration, and then each well was washed three times with 200 µL of ice cold 50 mM HEPES. The plate was allowed to dry overnight. 40 µL of scintillation cocktail (PerkinElmer, Waltham, MA, USA) was added to each well, and ^35^S activity was determined in a 1450 Microbeta plus microplate scintillation counter (PerkinElmer, Waltham, MA, USA) utilizing a one-minute counting time. The effect of the A_3_AR was determined by preincubating the membrane on ice with an A_3_AR antagonist (MRS 1334, 500 nM) or agonist (Cl-IB-MECA, 132 nM) for at least one hour before conducting the ^35^S-GTPγS binding experiment. 

To ask if the efficacy of CHA or the percentage of A1AR receptors in the high or low affinity state could explain the seasonal difference in response, Ki_Lo_ was determined by displacing 1 nM [^3^H] DPCPX with nine concentrations of CHA ranging between 100 pM and 10 µM in the presence of 100 µg/mL protein, 50 mM HEPES, 2 IU/mL ADA and 1 mM GTP. Ki_Hi_ was defined by displacing 1 nM [^3^H] DPCPX (PerkinElmer, Waltham, MA, USA) with CHA in the same manner as the Ki_Lo_ experiments but without GTP. The solution was allowed to equilibrate for 90 min at room temperature, as indicated by kinetic experiments. Free and bound [^3^H] DPCPX was separated through an Inotech glass fiber filter pad (0.35 mM thickness/0.75 µM retention) (Inotech Bio. Sys., Derwood, MD, USA) by rapid filtration (0.5 mL per sec per well) with a cell harvester (Tomtec, Hamden, CT, USA). The filter was then allowed to dry overnight. The next morning each well was isolated and placed in a scintillation vial. Scintillation cocktail (PerkinElmer, Waltham, MA, USA) was added, and radioactivity was determined (1450 Microbeta Plus, PerkinElmer, Waltham, MA, USA) with a five-minute count per well. Unlabeled agonists and antagonists were obtained from Sigma (St. Louis, MO, USA).

### 4.5. Data Analysis

^35^S-GTPγS specific binding was determined by subtracting non-specific binding from overall binding. Specific binding was converted to percent over constitutive receptor activity. pEC_50_, Hill slope and span were determined using the function Log(agonist) vs. response—variable slope (four parameters) in Graphpad Prism 5 (v 5.04) (Graphpad Software, La Jolla, CA, USA).

[^3^H] DPCPX bound was converted from cpm to fmol per mg protein, and the specific binding was calculated. A sum of squares F-test was used to determine if a one-site or two-site model was appropriate, and then the K_D_ and Bmax were calculated using the appropriate model using Graphpad Prism. Ki_Lo_ for the displacement of [^3^H] DPCPX in the presence of GTP was calculated using the average K_D_ (one or two site—Fit Ki). Ki_Hi_ was calculated for the displacement of [^3^H] DPCPX without GTP (one or two site—Fit Ki) using the average K_D_ and Ki_Lo_. Fraction Hi is the fraction of all the sites that have a high affinity for the competitor. It is calculated by Graphpad prism via the below equations.

Part1 = FractionHi * Span/(1 + 10^(X − LogEC50Hi))

Part2 = (1 − FractionHi) * Span/(1 + 10^(X − LogEC50Lo))

Data were analyzed by two-way ANOVA across brain region and season, followed by Tukey post-hoc comparisons or *t*-tests where indicated (R Studio). The significance threshold was defined as *p* < 0.05. Data are shown as mean ± SEM.

## Figures and Tables

**Table 1:** Characterization of CHA binding in summer and torpid AGS.

Region and Parameter Studied	Summer	Torpid
Forebrain		
[^3^H]DPCPX Displacement with CHA + GTP		
pKi_Lo_	6.77 ± 0.026	6.76 ± 0.020
[^3^H]DPCPX Displacement with CHA		
pKi_Hi_	8.56 ± 0.054	8.66 ± 0.024
Fraction High	0.41 ± 0.033	0.55 ± 0.024
pKi_Hi_/pKi_Lo_	1.26	1.28
Hippocampus		
[^3^H]DPCPX Displacement with CHA + GTP		
pKi_Lo_	6.42 ± 0.041	6.49 ± 0.074
[^3^H]DPCPX Displacement with CHA		
pKi_Hi_	8.82 ± 0.025	8.78 ± 0.021
Fraction High	0.51 ± 0.016	0.53 ± 0.020
pKi_Hi_/pKi_Lo_	1.37	1.35
Hypothalamus		
[^3^H]DPCPX Displacement with CHA + GTP		
pKi_Lo_	6.77 ± 0.030	6.84 ± 0.021
[^3^H]DPCPX Displacement with CHA		
pKi_Hi_	8.39 ± 0.013	8.39 ± 0.017
Fraction High	0.42 ± 0.066	0.55 ± 0.066
pKi_Hi_/pKi_Lo_	1.24	1.23

## Data Availability

The data presented in this study are available on request from the corresponding author.
